# Supplementary material for: Addressing disparity in attitudes and utilization of family planning among married couples in the pastoralist community of Fentale District, Eastern Ethiopia
Source: PLoS One. 2024 Sep 26;19(9):e0308633. doi: 10.1371/journal.pone.0308633 (PMC11426459; doi:10.1371/journal.pone.0308633)
Supplement: Supplementary file 1 [file pone.0308633.s001.docx]

BIIROO FAYYAA OROMIYAA
ኦሮሚያ ጤና ቢሮ
OROMIA HEALTH BUREAU

Guyyaa
Date
ቀን 24/06/2016 [illegible]

Lakk
Ref.no
ቁጥር [illegible]/2020

TO: SENA ADUGNA BEYENE

Subject: Ethical Approval of Research Protocol

Dear SENA ADUGNA BEYENE,

We are pleased to inform you that the Institutional Review Board (IRB) of the Oromia Regional Health Bureau has reviewed and granted ethical approval for your research protocol titled ***"Effect of Male Involvement in Family Planning Education on Contraceptive Use among the Reproductive Age Group in a Pastoralist Community of Fentale District."***

The IRB confirms that your research protocol meets the ethical standards outlined in both national and international guidelines. This signifies that your research has been ethically cleared and aligns with the established standards for conducting ethical research.This notification serves as an official confirmation of the ethical clearance for your research project. It underscores the importance of adhering to ethical standards and maintaining communication with the IRB should there be any significant changes to the approved research methodology.

Furthermore, the approval of your proposal on the influence of male participation in family planning education on contraceptive use among couples in a pastoralist community in Fentale District, Eastern Ethiopia, highlights the significance of ethical review and collaboration with relevant health organizations. This aligns with our broader goal of promoting informed and ethical health research practices in the Oromia region.

With regards,
Chairperson, IRB
Oromia Regional Health Bureau.

Gemechu Shume
Public Health Emergency
Management and Helath
Research Directorate Direct...

Biiroo Fayyaa Oromiyaatti Daayireektoreetii Hoogganaa B/T/Fayyaa Hawaasaa, Qorannoo fi Tajaajila B/Dhiiga
Oromia Health Bureau PHEM, Reserach & Blood Bank Service Directorate

Tel: +251993822229

Email:milkigeme@gmail.com

BIIROO FAYYAA OROMIYAA
ኦሮሚያ ጤና ቢሮ
OROMIA HEALTH BUREAU

Guyyaa ቀን Date 24/06/2013 EC
Lakk ቁጥር Ref.no BFO/17HBTFH/999/2020.

Title: **E**ffect of Male Involvement in Family Planning Education on Contraceptive Use among Reproductive Age Group in Pastoralist Community of Female DistricName of the Applicant: Mr **Sena Adugna** Application Number: OHB **/IRB/20_BEFO/HBTFH/999/2020)** Date Received: **24/06/2013 EC**

Covering Letter	✓
Submission Check List	✓ ✓
Declaration of Applicant	✓
One-page summary proposal	✓
Detailed research proposal	✓
All study instruments in English (questionnaires/interview guides/checklist/data extraction forms) with Afan Oromo and translations where relevant	✓
Information sheet in English	✓
Consent forms in English with Afan Oromo	✓
Any other relevant documents in English and relevant Curriculum vitae of all investigators	✓
Approval letter from the relevant Board of Study (if applicable)	✓

6955
befokom2008@gmail.com
info@orhb.gov.et
www.orhb.gov.et
Oromia health bureau
t.me/ORHBCommunication
Saarbet (Calcalii) Finfinnee
